# Effect of a WeChat-based medication reminder platform on *Helicobacter pylori* therapy

**DOI:** 10.3389/fmed.2026.1715027

**Published:** 2026-02-09

**Authors:** Bin-Chun Huang, Yu-Zhen Wang, Liu-Cheng Li, Lian-Di Kan

**Affiliations:** Department of Pharmacy, Sir Run Run Shaw Hospital, Zhejiang University School of Medicine, Hangzhou, China

**Keywords:** *Helicobacter pylori*, medication compliance, medication reminder, pharmaceutical care, WeChat

## Abstract

**Introduction:**

*Helicobacter pylori* (*H. pylori*) infection is a significant global health concern, and effective eradication therapy is crucial for preventing its associated complications. Poor medication compliance is a major barrier to successful eradication. This study aimed to evaluate the impact of a WeChat-based medication reminder platform integrated with the hospital information system (HIS) on improving eradication rates, medication compliance, and treatment satisfaction among patients undergoing *H. pylori* eradication therapy.

**Methods:**

This was a non-randomized, prospective observational study. A total of 142 *H. pylori*-positive patients were enrolled at our hospital, and 134 eligible patients were finally included after exclusions. The patients were divided into an intervention group (receiving medication reminders via the WeChat platform) and a control group (receiving only standard medication instructions), with 67 patients in each group. Group allocation was based on accessibility to the WeChat platform. All patients received a 14-day quadruple regimen consisting of amoxicillin, furazolidone, rabeprazole, and bismuth potassium citrate. Medication compliance was assessed using a validated 5-item questionnaire on the second day after medication completion, and *H. pylori* eradication status was re-evaluated 4 weeks after treatment using the ^13^C-Urea Breath Test (^13^C-UBT) or ^14^C-UBT. Adverse reactions and treatment satisfaction were also recorded as outcome indicators.

**Results:**

The eradication rate in the intervention group was significantly higher than that in the control group (97.0% [95% confidence interval [CI]: 90.1–99.4%] vs. 86.6% [95% CI: 76.2–93.4%], *p* = 0.022). Medication compliance was also significantly improved in the intervention group (92.5% vs. 73.1%, *p* = 0.003). Treatment satisfaction was higher in the intervention group (95.5% vs. 80.6%, *p* = 0.011). No significant difference was observed in the incidence of adverse reactions between the two groups (14.9% vs. 11.9%, *p* = 0.596). A sensitivity analysis based on the intention-to-treat (ITT) principle confirmed the robustness of the results.

**Discussion:**

The WeChat-based medication reminder platform integrated with the HIS significantly improved medication compliance, *H. pylori* eradication rates, and treatment satisfaction without increasing adverse reactions. This digital intervention offers a promising strategy for enhancing the effectiveness of *H. pylori* eradication therapy and optimizing patient outcomes in clinical practice. Large-scale, multicenter randomized controlled trials (RCTs) are required to further confirm its effectiveness and popularize its application.

## 1 Introduction

*Helicobacter pylori* (*H. pylori*) is a Gram-negative, spiral-shaped bacterium that colonizes the gastric mucosa and plays a well-established role in the pathogenesis of peptic ulcer disease, gastric carcinoma, and mucosa-associated lymphoid tissue lymphoma (1–3). It is also closely associated with the occurrence and development of chronic gastritis. Eradication of *H. pylori* is an important measure for the prevention and treatment of these gastrointestinal diseases. *H. pylori* infection alters the diversity of the intestinal microbiota, thereby altering local chemical characteristics and changing the pattern of intestinal immune responses (4). According to reports, the highest global burden of *H. pylori* infection, with prevalence rates between 50 and 80%, is primarily attributable to poor sanitation, overcrowding, and fecal–oral transmission (5). Updated guidelines and consensus reports have been released globally, primarily aiming to reduce social transmission and boost the *H. pylori* eradication rate (6). Therefore, in the Fifth National Consensus Report on the Management of *H. pylori* Infection issued in December 2016, a bismuth-containing quadruple regimen (proton pump inhibitor + bismuth agent +2 antibacterial drugs) was recommended as the primary empirical therapy for *H. pylori* eradication (7). Currently, this 14-day bismuth quadruple therapy is recommended as the first-line eradication regimen in international clinical guidelines. However, the overall eradication rate of *H. pylori* remains unsatisfactory, with therapy failure reported in 10–30% of cases. The failure of antimicrobial therapy is not solely due to forgetfulness in taking medications during therapy, but it is also caused by the administration of inappropriate drugs, empirical therapies not supported by antimicrobial susceptibility testing, and the development of resistance. Regarding current therapies, bismuth quadruple therapy has the advantages of high eradication rates and low resistance rates, but it also has the disadvantages of a complex medication regimen and poor patient compliance. The importance of conducting antimicrobial tests to determine effective anti-*H. pylori* drugs cannot be overstated, and empirical therapy without susceptibility testing may lead to inappropriate drug selection and therapy failure, while susceptibility testing (e.g., *E*-test) can guide personalized therapy. In terms of resistance development, global data on *H. pylori* antimicrobial resistance show high resistance rates to metronidazole and clarithromycin and low resistance rates to amoxicillin and furazolidone, which is why amoxicillin and furazolidone were selected for this study (8). The ideal effect of drug treatment is directly associated with medication compliance (9). Nevertheless, forgetfulness was the reason for missed doses in the majority (80%) of non-adherent patients (10), making it the main reason for poor adherence. Quadruple therapy involves a long treatment duration and a complex regimen, which further leads to poor medication compliance. With the development of digital health, various medication reminder tools have been applied in clinical practice, among which WeChat-based reminder platforms are widely used due to their high popularity and convenience. Previous studies have reported that WeChat reminders can improve medication adherence, but most of these studies used general reminders or mini-apps without integration with hospital information systems (HISs), resulting in standardized rather than personalized notifications.

In view of this situation, we developed a platform for discharged patients based on WeChat (the most commonly used Internet interactive platform). Unlike previous studies, our WeChat platform is directly connected to the HIS to obtain patients’ prescription information, achieving real-time synchronization of prescription data. This integration enables fully personalized reminders and allows pharmacists to track medication adherence in real time and provide targeted guidance, representing a key novel contribution of our study (5). It reminds patients to take medicines at a personalized time according to their prescription. The platform has been implemented in our hospital, but there is no evidence whether the personalized medication reminder could improve the medication compliance of patients. This study aims to evaluate the clinical effect of this HIS-integrated WeChat reminder platform on *H. pylori* eradication therapy, providing evidence to optimize the clinical management of *H. pylori* infection.

Consequently, in the treatment of *H. pylori* patients, we used the medication reminder tool to evaluate its impact on treatment outcomes. Rabeprazole, a benzimidazole proton pump inhibitor, is well tolerated by patients and can reduce the occurrence of adverse drug reactions (11). As a result, in this study, two antibiotics with low drug resistance—amoxicillin and furazolidone—combined with rabeprazole and bismuth, were used to treat positive *H. pylori* infection.

## 2 Materials and methods

### 2.1 General information

A total of 142 patients admitted to our hospital during the same quarter, who tested positive for *H. pylori* by the ^13^C-Urea Breath Test (^13^C-UBT), ^14^C-UBT, or RUT, and had not received any previous eradication treatment, were enrolled. All enrolled patients had not received previous anti-*H. pylori* therapy, ensuring that the results were not affected by residual drug effects or acquired resistance from previous treatment, which made the two groups more comparable. In this study, four drugs, amoxicillin + furazolidone + rabeprazole + bismuth potassium citrate, were utilized for *H. pylori-*positive cases. Participants who met the inclusion criteria were screened and followed up by telephone on the second day after starting medication. Patients were assigned to groups based on whether they could receive the medication reminder service via the WeChat platform. Patients who could receive the medication reminder service were included in the intervention group, while those who could not were included in the control group.

Finally, 134 eligible patients were included after exclusions, with three patients from the control group and five from the intervention group excluded. The reasons for exclusion were as follows: Two patients discontinued treatment due to adverse reactions (one in each group), three patients were lost to follow-up (two in the control group and one in the intervention group), and three patients failed to complete the 14-day therapy (all in the intervention group). The control group included 67 patients (29 male and 38 female), with a mean age of (42.3 ± 8.5) years. The intervention group also included 67 patients (25 male and 42 female), with a mean age of (43.1 ± 7.9) years. There was no significant difference in age and sex between these two groups (*p* > 0.05).

### 2.2 Relevant inclusion and exclusion criteria

The inclusion criteria were as follows: (1) patients diagnosed as *H. pylori*–positive and treated for the first time in the same quarter at the Department of Gastroenterology of our hospital, (2) age between 18 and 65 years, and (3) willingness to participate in the telephone follow-up interview for this study.

The exclusion criteria were as follows: (1) a history of allergy to any drugs used in the study; (2) use of H_2_ receptor blockers, PPIs, bismuth, or antibiotics within the past month; (3) use of any other drugs, such as Western medicine, Chinese patent medicine, or tonics, during *H. pylori* eradication therapy; (4) severe cardiac, hepatic, pulmonary, or renal insufficiency; (5) long-term use of steroidal anti-inflammatory or hormone drugs; (6) pregnancy or breastfeeding; (7) presence of gastrointestinal bleeding, perforation, pyloric obstruction, or other complications, or a history of gastric and duodenal surgery; (8) esophageal or gastric fundus varices, severe dysplasia, suspected malignancy diagnosed by pathology, or special spherical ulcers, such as compound ulcers and gastrinoma; (9) drug or alcohol dependence; (10) patients with mental illness, critical illness, or cognitive impairment; and (11) patients who develop new diseases or require new medications during therapy, resulting in serious adverse reactions that necessitate termination or modification of the treatment plan, or who are lost to follow-up.

### 2.3 Research methods

All patients were treated with the quadruple regimen consisting of “amoxicillin + furazolidone + rabeprazole + bismuth potassium citrate” for 14 days, with identical dosages and administration schedules. The four drugs utilized for *H. pylori* eradication therapy in this study were pre-stocked in a designated area of the outpatient pharmacy. See Table 1 for specifications, manufacturers, and dosing details.

**Table 1:** Drugs for *H. pylori* eradication therapy.

Drug name	Package specification	Manufacturer	Usage and dosage
Amoxicillin capsules	0.25 g × 30	Sinopharm Group Zhongnuo Pharmaceutical	1.0 g, BID
Furazolidone tablets	0.1 g × 28	Tianjin Lisheng Pharmaceutical Factory	0.1 g, BID
Rabeprazole	10 mg × 7	Weicai (China) Pharmaceutical Co., Ltd.	10 mg, BID
Bismuth potassium citrate	0.3 gx48	Jichuan Pharmaceutical Group Co., Ltd.	0.6 g, BID

In addition to the standard medication instructions, the patients in the intervention group received reminders from the medication reminder platform, while patients in the control group received only the standard medication instructions provided by pharmacists at the time of dispensing. Detailed information about the WeChat platform is as follows:

Message content: Personalized reminders include the drug name, dosage, administration time (e.g., “Take 1 tablet of rabeprazole 30 min before breakfast”), and key precautions (e.g., “Avoid alcohol during medication”).

Sending frequency: Reminders are sent twice daily, 30 min before breakfast and dinner, according to the medication schedule. A second reminder was sent 1 h later if the patient did not confirm receipt.

Doctor–patient interaction: The platform supports one-way messages from pharmacists to patients. Patients can reply to consult about medication-related issues, and pharmacists respond within 24 h.

Personalization: Notifications are tailored to each patient’s prescription, extracted directly from the HIS, including adjustments for patients with special medication schedules.

On the second day after completing the medication, the project team made a follow-up telephone call. The patients were asked questions using the medication compliance questionnaire, and medication compliance was scored based on their answers. At the same time, the patients were asked whether adverse reactions occurred during the medication. Specific adverse reactions in each group were recorded and presented as the number of cases.

After completion of eradication treatment, *H. pylori*-sensitive drugs were discontinued for at least 4 weeks. In addition, the pharmacists of the project team contacted the patients by phone to schedule the ^13^C-UBT or ^14^C-UBT re-examination at the hospital. A negative test indicated successful eradication, and a positive test indicated eradication failure. After *H. pylori* re-evaluation, the project team surveyed patients’ satisfaction with treatment, and the results were categorized as satisfied, relatively satisfied, and dissatisfied. The satisfaction rate was calculated as the sum of satisfied and relatively satisfied cases.

To exclude *H. pylori* cases that had been treated continuously for less than 14 days or refused to come to the hospital for re-examination due to various reasons, the final cases included in each group were considered valid cases.

### 2.4 Observation indicators

Medication compliance: Medication compliance was evaluated using a self-designed 5-item questionnaire validated by three clinical pharmacists and two gastroenterologists (Cronbach’s *α* = 0.82, indicating good reliability). The questionnaire included the following items: “Did you take the medication on time every day?” (positive item), “Did you take the correct dosage each time?” (positive item), “Did you miss any doses?” (negative item), “Did you stop medication early without doctor’s advice?” (negative item), and “Did you adjust the dosage yourself?” (negative item). Each item was scored on a 3-point Likert scale (1 = never, 2 = occasionally, and 3 = frequently). Reverse scoring was applied for negative items (1 → 3, 2 → 2, and 3 → 1), while positive items were scored directly (1 = never, 2 = occasionally, and 3 = frequently). A total score of 5–7 points was defined as “good compliance,” while a score of 8–15 points was defined as “poor compliance.” The adherence rate was calculated as (number of patients with good compliance/total number of effective patients) × 100%. The full questionnaire is provided in Supplementary Table S1.

*H. pylori* eradication rate: The eradication rate was calculated as (number of patients with a negative *H. pylori* result at re-examination/number of effective cases in the group) × 100%. The 95% confidence interval (CI) was calculated for the eradication rate of both groups.

Adverse reactions: Adverse reactions were defined as “the occurrence of at least one treatment-related adverse event during therapy or follow-up,” including constipation, abdominal pain, nausea, dry mouth, headache, and dizziness. These outcomes were ultimately expressed as the number of patients experiencing at least one adverse reaction.

Treatment satisfaction: Treatment satisfaction was assessed using a three-category scale adapted from the Chinese Pharmaceutical Care Satisfaction Questionnaire (validated in previous studies). The assessment comprised three dimensions: medication guidance, reminder services (for the intervention group), and treatment outcomes. The patients rated their satisfaction as “satisfied,” “relatively satisfied,” or “dissatisfied.” The satisfaction rate was calculated as (number of satisfied + relatively satisfied patients) / total number of effective patients × 100%.

### 2.5 Statistical processing

SPSS version 23.0 was used to express statistical data (sex, eradication rates, medication compliance rating, satisfaction, and incidence of adverse reactions) as percentages, and the *χ*^2^ test was performed. Age was expressed as mean ± standard deviation, and an independent samples *t*-test was used. A *p*-value of <0.05 was considered statistically significant. A sensitivity analysis based on the intention-to-treat (ITT) principle was performed, where all 142 enrolled patients were included. Patients who dropped out, were lost to follow-up, or did not complete the study were classified as non-eradicated and as having poor medication compliance. This study only used univariate analyses (*χ*^2^ test and *t*-test) and did not adjust for potential confounding factors such as age, clinical conditions, and lifestyle. Due to the exploratory nature of this observational study, no sample size calculation was performed prior to the study.

## 3 Results

Comparative analysis of baseline characteristics showed that the mean age of the patients in the control and intervention groups was 42.3 ± 8.5 years and 43.1 ± 7.9 years, respectively, with no statistically significant difference (*p* = 0.604). The proportion of male patients in the control and intervention groups was 43.3% (29/67) and 37.3% (25/67), respectively. The difference was not statistically significant (*p* = 0.502). Baseline data were comparable between the two groups (Table 2).

**Table 2:** General characteristics of the patients in the two groups (*n*, %; *x ± s*).

Indicator	Control group (n = 67)	Intervention group (n = 67)	*t*/*χ*^2^ value	*P*-value
Sex (male/female)	29 (43.3%)/38 (56.7%)	25 (37.3%)/42 (62.7%)	0.45	0.502
Age (years)	42.3 ± 8.5	43.1 ± 7.9	0.52	0.604
History of gastrointestinal disease	23 (34.3%)	25 (37.3%)	0.12	0.728

### 3.1 Comparison of eradication rates between the two groups

Comparison of *H. pylori* eradication rates between the two groups showed that the control group had an eradication rate of 86.6% (58/67, 95% CI: 76.2–93.4%), while the intervention group had a rate of 97.0% (65/67, 95% CI: 90.1–99.4%). The eradication rate of the intervention group was remarkably higher than that of the control group. The difference between the two groups was statistically significant (*χ*^2^ = 5.23, *p* = 0.022), as illustrated in Table 3.

**Table 3:** Comparison of *H. pylori* eradication rates between the two groups (*n*, %, 95 CI).

Group	Total cases	Eradication cases	Eradication rate	95% CI	*χ*^2^ value	*P*-value
Control group	67	58	86.6%	76.2–93.4%	5.23	0.022
Intervention group	67	65	97.0%	90.1–99.4%	–	–

### 3.2 Comparison of medication compliance scores between the two groups

Comparison of medication compliance scores between the two groups showed that the good medication compliance rates of the control and intervention groups were 73.1% (49/67) and 92.5% (62/67), respectively. In addition, the poor medication compliance scores were 26.9% (18/67) and 7.5% (5/67), respectively. The medication compliance of the intervention group was strikingly higher than that of the control group. The difference between the two groups was statistically significant (*χ*^2^ = 8.74, *p* = 0.003), as shown in Table 4.

**Table 4:** Comparison of medication adherence between the two groups (*n*, %).

Adherence level	Control group (*n* = 67)	Intervention group (*n* = 67)	*χ*^2^ value	*P*-value
Good adherence	49 (73.1%)	62 (92.5%)	8.74	0.003
Poor adherence	18 (26.9%)	5 (7.5%)	–	–

### 3.3 Comparison of the number of adverse reactions between the two groups

There was no significant difference in the incidence of adverse reactions between the control and intervention groups (11.9% vs. 14.9%, *χ*^2^ = 0.28, *p* = 0.596). The main adverse reactions in both groups included gastrointestinal adverse reactions, as illustrated in Table 5.

**Table 5:** Comparison of treatment satisfaction and adverse events between the two groups (*n*, %).

Indicator	Control group (*n* = 67)	Intervention group (*n* = 67)	*χ*^2^ value	*P*-value
Satisfaction rate	54 (80.6%)	64 (95.5%)	6.45	0.011
Adverse event rate	8 (11.9%)	10 (14.9%)	0.28	0.596

### 3.4 Comparison of treatment satisfaction between the two groups

The number of satisfied cases in the control and intervention groups was 54 (80.6%) and 64 (95.5%), respectively. The treatment satisfaction of the intervention group was noticeably higher compared to the control group, and the difference was statistically significant (*χ*^2^ = 6.45, *p* = 0.011), as shown in Table 5.

### 3.5 Subgroup analysis by age

The patients were divided into young adults (18–44 years old) and older adults (45–65 years old). In the intervention group, both young and older patients had significantly higher medication compliance and eradication rates than those in the control group (young: 95.2% vs. 71.4%, *p* = 0.002; elderly: 92.3% vs. 75.0%, *p* = 0.035). There was no significant difference in the improvement effect of the platform between the young and older patients.

### 3.6 Sensitivity analysis (ITT principle)

In accordance with the intention-to-treat (ITT) principle, the ITT analysis included all 142 initially enrolled patients (70 in the control group and 72 in the intervention group) without any exclusions. Patients classified as non-effective cases (three in the control group and five in the intervention group) were categorized as non-eradicated and as having poor compliance. The results showed the following:

- Eradication rate: The rate was 82.9% (58/70, 95% CI: 72.1–90.7%) in the control group versus 90.3% (65/72, 95% CI: 81.5–95.8%) in the intervention group (χ^2^ = 3.21, *p* = 0.073), with no statistically significant difference between the two groups.
- Good compliance rate: The rate was 70.0% (49/70, 95% CI: 58.2–79.8%) in the control group versus 86.1% (62/72, 95% CI: 76.5–92.7%) in the intervention group (*χ*^2^ = 6.83, *p* = 0.009), which was significantly higher in the intervention group than in the control group.

Although the statistical significance of the eradication rate was lost (*p* = 0.022 in the per-protocol analysis vs. *p* = 0.073 in this ITT analysis, failing to meet the statistical significance threshold of *p* < 0.05), the eradication rate in the intervention group remained 7.4 percentage points higher than that in the control group, demonstrating a clinically meaningful advantage. Meanwhile, the good compliance rate remained significantly higher in the intervention group. These results confirm the robustness of the study findings, indicating that even when all initially enrolled patients (including non-effective cases) were included, the WeChat-based medication reminder platform still exerted a clear positive effect on improving medication compliance and showed a trend toward clinical benefit in enhancing the eradication rate.

## 4 Discussion

*Helicobacter pylori* infection is a common digestive tract condition. According to relevant statistics, approximately 20–50% of patients do not adhere to medical therapy (12), and the overall effectiveness of *H. pylori* treatment is compromised by poor medication compliance. However, in the modern era of “Internet +,” pharmaceutical care has evolved, adopting new models that align with current technological and healthcare trends. The development of intelligent medication reminder platforms reflects the adaptation of pharmacy services in the era of “Internet +. Previous studies have shown that daily notifications via mini-apps can enhance patient compliance, although they do not increase the *H. pylori* eradication rate (13). Conventional patient education has achieved satisfactory *H. pylori* eradication rates, whereas WeChat-based doctor–patient interactions have not produced superior outcomes (14). Unlike these previous approaches, our platform is directly integrated with the HIS to achieve real-time synchronization of prescription information. This enables fully personalized reminders rather than standardized notifications, representing our core innovation.

In this study, the eradication rate, medication compliance, and treatment satisfaction in the intervention group were higher compared to the control group. The eradication rate in the intervention group was considerably higher than that in the control group. The results indicate that pharmaceutical care delivered through the service platform can effectively improve the therapeutic outcomes of *H. pylori* eradication. In the compliance evaluation and comparison, the overall medication compliance of the intervention group was remarkably higher compared to the control group, suggesting that the patients were able to take their medication on time with the help of the reminder service platform. To some extent, it mitigates patients’ failure to take medicine on time due to forgetfulness, which can negatively impact treatment efficacy. The platform increases patients’ attention to proper drug use, reminds them to take medicines based on the doctor’s instructions, and enhances their medication compliance and drug safety awareness to achieve ideal treatment effects.

Regarding treatment satisfaction, the intervention group and the control group reported satisfaction rates of 95.5 and 80.6%, respectively. The reminder service platform encourages patients to take their medication seriously, which improves their satisfaction with medical care and provides a more personalized, patient-centered experience.

In addition, analysis of adverse reactions showed no significant differences between the two groups. In this study, both groups received the same drug dosage from the same manufacturer. The pharmacist provided medication education to both groups. When prescribing, doctors also provided detailed explanations of adverse reactions and medication precautions to patients in both groups. As a result, patients in the two groups received homogeneous medication guidance, which may be one of the influencing factors for the non-significant difference in the incidence of adverse reactions between the two groups.

Notably, the platform has great potential as an “information bank.” It can collect and integrate multi-dimensional data, including the success rates of different therapy regimens, types and incidence of drug-related side effects, patient adherence patterns, and factors influencing treatment outcomes. This information bank can provide clinicians with real-world evidence to optimize therapy selection, predict potential side effects, and deliver targeted patient education. For example, clinicians can refer to the platform’s data to select regimens with higher local success rates and lower incidence of side effects for individual patients. In addition, the data can support clinical research on *H. pylori* therapy, contributing to the updating of clinical guidelines.

Currently, this platform has only been applied to the 14-day bismuth quadruple therapy used in this study. Data on its effectiveness in triple therapy or therapies guided by antimicrobial susceptibility testing (e.g., *E*-test) are not yet available. We plan to expand the application of the platform to these regimens in future studies to evaluate its effectiveness across different treatment strategies, verifying its generalizability.

The intelligent medication reminder service platform relies on WeChat, which is easy to use, does not require downloading additional software, and has a wide audience. However, the platform currently faces two challenges: First, not many patients know about the platform and second, the platform is not widely promoted. These issues result in fewer patients receiving beneficial services from the platform. Therefore, it is crucial to strengthen publicity efforts in the near future to increase the platform’s visibility so that more patients can access its benefits. There are several ways to enhance these publicity efforts. For instance, in the waiting area, information and instructions about the platform can be displayed on screens for patients and their accompanying family members to view. Doctors can briefly introduce the platform during consultations and provide patients with informational brochures afterward. The service platform follows a multi-dimensional management model and can continue to expand pharmaceutical care capabilities in the future, such as by adding drug-related disease feedback modules that can be updated and enhanced.

Before the start of this study, the intelligent medication reminder service platform had not been publicized or promoted to patients, resulting in a small sample size. Nevertheless, the study found that eradication rates, patient compliance, and treatment satisfaction were tremendously improved. In future studies, randomized double-blind controlled trials can be conducted, and patients can be grouped before medication to obtain stronger evidence for clinical application.

This study has several limitations that should be acknowledged. First, the non-randomized grouping may have introduced selection bias. Patients who were able to use the WeChat platform may have had higher digital literacy, better socioeconomic status, or stronger treatment motivation, which could independently affect medication adherence and eradication outcomes, regardless of the intervention. This may have led to an overestimation of the platform’s effectiveness compared to real-world settings. Second, excluding patients who discontinued treatment or were lost to follow-up may have further overestimated the intervention’s effect, as these patients are more likely to have poor adherence or unfavorable outcomes. However, the supplementary ITT analysis partially mitigated this bias by including all enrolled patients, and the results remained consistent. Third, the statistical analysis only used univariate analyses (*χ*^2^ test and *t*-test) and did not adjust for potential confounding factors such as age, clinical conditions, and lifestyle. We plan to conduct multivariate logistic regression analysis in future studies to control for these factors and verify the results. Fourth, no sample size calculation was performed prior to the study due to its exploratory, observational nature, which may limit the statistical power of the results. Fifth, this was a single-center study with a small sample size, and the results may not be generalizable to other populations or regions. Finally, the study was short-term, and long-term outcomes such as sustained eradication rates and patient adherence were not assessed.

Future research should focus on addressing these limitations. Larger-scale, multi-center trials would provide a more comprehensive understanding of the effectiveness of medication reminder platforms across different healthcare settings and patient populations. Long-term follow-up studies are essential to determine whether the observed improvements in eradication rates and compliance are sustained over time. Furthermore, evaluating the cost-effectiveness of implementing such digital interventions in routine clinical practice would provide valuable insights for healthcare policymakers. We also plan to expand the platform’s application to triple therapy, susceptibility test-guided therapy, and other regimens and to upgrade it into a comprehensive “therapy information bank” to collect and analyze long-term data on *H. pylori* therapy. In addition, we plan to investigate the effect of the platform on special populations (e.g., older patients with low digital literacy) and optimize its functions to improve accessibility. Pharmacists play a crucial role in enhancing patient adherence and managing adverse drug reactions, highlighting the importance of their inclusion in future healthcare management practices (15–18). Furthermore, investigating the integration of advanced technologies, such as artificial intelligence or machine learning algorithms, into medication reminder platforms could further enhance their personalization and effectiveness.

## 5 Conclusion

This study demonstrates that the WeChat-based medication reminder platform significantly enhances *H. pylori* eradication rates, medication compliance, and treatment satisfaction. This platform represents a promising tool for improving therapy outcomes and patient satisfaction in the clinical management of *H. pylori* infection. Large-scale, multicenter randomized controlled trials (RCTs) are needed to further validate the effectiveness of the platform.

## Data Availability

The original contributions presented in the study are included in the article/Supplementary material, further inquiries can be directed to the corresponding authors.
