# Supplementary material for: Effect of a WeChat-based medication reminder platform on Helicobacter pylori therapy
Source: Front Med (Lausanne). 2026 Feb 9;13:1715027. doi: 10.3389/fmed.2026.1715027 (PMC12926105; doi:10.3389/fmed.2026.1715027)
Supplement: Supplementary file 1 [file Table_1.docx]

**Supplementary Table S1. Medication Compliance Questionnaire**

| **Item Number** | **Questionnaire Item** | **Item Type** | **Original Scoring (1–3 Scale)** | **Scoring Rule (Adjusted)** |
| --- | --- | --- | --- | --- |
| 1 | Did you take the medication on time every day? | Positive | 1 = Never (fully on time)  2 = Occasionally (missed a few days)  3 = Frequently (missed multiple days) | Direct scoring:  1 point → 1 point  2 points → 2 points  3 points → 3 points |
| 2 | Did you take the correct dosage each time? | Positive | 1 = Never (dosage fully correct)  2 = Occasionally (a few dosage errors)  3 = Frequently (multiple dosage errors) | Direct scoring:  1 point → 1 point  2 points → 2 points  3 points → 3 points |
| 3 | Did you miss any doses? | Negative | 1 = Never (no missed doses)  2 = Occasionally (a few missed doses)  3 = Frequently (multiple missed doses) | Reverse scoring:  1 point → 3 points  2 points → 2 points  3 points → 1 point |
| 4 | Did you stop medication early without doctor’s advice? | Negative | 1 = Never (no early discontinuation)  2 = Occasionally (temporary early discontinuation)  3 = Frequently (prolonged early discontinuation) | Reverse scoring:  1 point → 3 points  2 points → 2 points  3 points → 1 point |
| 5 | Did you adjust the dosage yourself? | Negative | 1 = Never (no dosage adjustment)  2 = Occasionally (minor dosage adjustment)  3 = Frequently (multiple dosage adjustments) | Reverse scoring:  1 point → 3 points  2 points → 2 points  3 points → 1 point |

Total Score Calculation and Compliance Classification

1.Total Score: Sum the adjusted scores of all 5 items (total score range: 5–15 points).

2.Compliance Classification:

- Good compliance: 5–7 points (Minimal or no deviations from the medication regimen).
- Poor compliance: 8–15 points (Significant deviations, such as frequent missed doses, incorrect dosage, or early withdrawal).

Questionnaire Validation

- Validated by 3 clinical pharmacists and 2 gastroenterologists.
- Cronbach’s α coefficient = 0.82, indicating good internal consistency and reliability.
- Administered via telephone interview on the second day after completion of the 14-day H. pylori eradication therapy.
